# Extra-pontine Myelinolysis After Rapid Correction of Hyponatremia Responding to Levodopa

**DOI:** 10.7759/cureus.52707

**Published:** 2024-01-22

**Authors:** Jagannath Dhadwad, Anish Chitnis

**Affiliations:** 1 General Medicine, Dr. D. Y. Patil Medical College, Hospital and Research Centre, Dr. D. Y. Patil Vidyapeeth, Pune, IND

**Keywords:** extra-pontine myelinolysis (epm), oral levodopa, osmotic demyelination syndrome (ods), secondary parkinsonism, hypovolemic hyponatremia

## Abstract

Osmotic demyelinating disease of the central nervous system has two variants: central pontine myelinolysis and extra-pontine myelinolysis (EPM). Up to 10% of cases of osmotic demyelination syndrome are associated with EPM, which mostly affects the thalamus and basal ganglia. It is commonly associated with the rapid correction of hyponatremia. An elderly woman in her 60s presented with complaints of acute gastroenteritis and giddiness and visited the emergency ward. On examination, she was conscious and oriented to time but disoriented to place and person and had slurring of speech with signs of dehydration. Her serum sodium levels were 100 meq/L, and her brain MRI was normal. After shifting her to the intensive care unit, she was treated with 200 ml of 3% NaCl bolus to correct her hyponatremia. On day three, she began developing rigidity in both lower limbs, which progressed to the upper limbs with hyperreflexia and mutism. A brain MRI was done, which showed subtle hyperintensities in the caudate lobe with no other new findings. Her serum aldosterone and cortisol were on the lower side of the normal range. Treatment of tablet levodopa-carbidopa (100/25) combination thrice a day (TDS) led to an improvement in her health condition.

## Introduction

This article reports a case of parkinsonism after a rapid correction of hyponatremia, which is known as extra-pontine myelinolysis (EPM), a type of osmotic demyelination syndrome (ODS). It is characterized by extrapyramidal signs such as mutism, rigidity, bradykinesia, and hyperreflexia after the rapid correction of hyponatremia with intravenous 3% NaCl (hypertonic saline). Up to 10% of cases of ODS may involve EPM, primarily affecting the thalamus and basal ganglia [1]. It is commonly associated with the rapid correction of hyponatremia with intravenous 3% NaCl (hypertonic saline). It is unclear exactly what causes ODS.

## Case presentation

We report a case of a previously healthy 55-year-old female who presented to the emergency department with complaints of loose stools for four days that were watery in consistency, non-foul smelling, and non-mucoid. The patient reported four to five episodes per day, which were associated with crampy pain in the abdomen. Moreover, she had three to four episodes per day of nausea and vomiting that were non-projectile and contained food particles. She complained of low-grade fever for four days, which was associated with generalized weakness. She had a history of pulmonary tuberculosis 20 years ago, for which she took antituberculosis treatment for six months. There was no history of hypertension, diabetes mellitus type 2, bronchial asthma, ischemic heart disease, chronic kidney disease, or stroke.

On the day of admission, the patient had an episode of giddiness in the outpatient department, during which she suddenly fell. She was transferred to the emergency department immediately. In the emergency ward, she was oriented to time but was disoriented to place and person. Moreover, she had a slurring of speech after the episode. She had no complaints of loss of consciousness, involuntary movements of limbs, or incontinence of bowel or bladder. There was no chest pain, palpitations, or breathlessness.

On examination, the patient was oriented to time but disoriented to place and person. The patient was dehydrated, as her tongue was dry. There were no contusions or abrasions on the head or the body. On general examination, her pulse was 100 beats/min, blood pressure was 130/80 mmHg, respiratory rate was 20/min, and SpO2 was 98% in room air. In the central nervous system examination, the patient had a normal tone in all four limbs, with deep tendon reflex 2+ and power grade 4/5 at all joints. There was normal coordination and no nystagmus. In the respiratory system, there was decreased breath sound on the left side with coarse crepitations.

Laboratory findings on day one were as follows: hemoglobin was 10 g/dL; white blood cells, platelets, and renal function tests were in the normal range; however, sodium was <100 mmol/L, and chloride was 70 mmol/L. A chest x-ray showed a collapse of the left lung with a mediastinal shift to the left side. An electrocardiogram showed normal sinus rhythm with normal PR interval and ST-T changes. Her brain MRI (plain) findings were chronic ischemic changes with age-related cortical atrophy. High-resolution computerized tomography of the thorax showed the near collapse of the left lung with a mediastinal shift to the left side with the possibility of recurrence of the previous infection. Fundus and cranial nerve examination was normal. She was immediately shifted to the medical intensive care unit and was given two boluses of 3% NaCl (hypertonic saline), and intravenous antibiotics (ceftriaxone 1 gm BD and metronidazole 100 mL thrice a day (TDS)) were started.

Laboratory findings on day two were as follows: serum sodium rose to 104 mmol/L and chloride to 97 mmol/L. On day three, the patient developed rigidity in the lower limbs, which progressed to the upper limbs, and hyperreflexia (deep tendon reflex 3+) with mask-like facies and mutism. Serum cortisol (morning sample) was low, and serum aldosterone was on the lower side of the normal range (i.e., serum cortisol was 3.30 μg/L and serum aldosterone 2.95 ng/L).

On day three, she was started on hydrocortisone tablets 10 mg TDS, which improved her sodium levels with no improvement in Parkinsonian features. On the same day, a brain MRI was performed followed by contrast-enhanced computerized tomography of the abdomen/pelvis, which showed no changes of adrenal hemorrhage or any other adrenal tubercular changes. Sputum acid-fast bacilli (AFB) and cartridge-based nucleic acid amplification test (CBNAAT) were negative.

**Figure 1:**
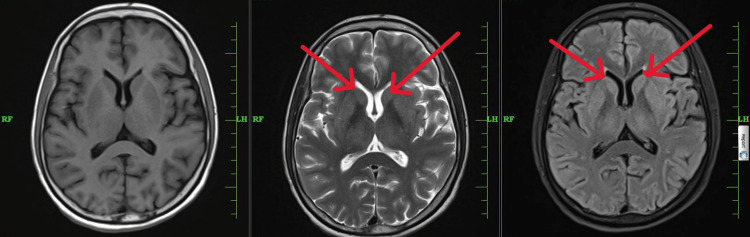
T1- and T2-weighted MRI of the brain shows subtle hyperintensities in the bilateral caudate lobe (red arrows) MRI: Magnetic resonance imagining; A: T1-weighted MRI of the brain; B: T2-weighted MRI of the brain; C: T2 FLAIR MRI of the brain

On day five, MRI findings showed subtle hyperintensities in the caudate lobe (Figure 1) with no other new findings. The patient was started on levodopa + carbidopa 100/25 mg combination tablets, which showed dramatic improvement in five days, and she was later discharged.

## Discussion

ODS comprises two variants, namely, central pontine myelinolysis and EPM, whose features are quadriparesis, dysphagia, dysarthria, locked-in syndrome, coma, and, rarely, movement disorders [2]. Hypertonic stress with rapid correction of hyponatremia causes apoptosis in astrocytes and disruption of the blood-brain barrier.

Our case report presents a case of EPM with a rapid correction of hyponatremia, in contrast to ODS developing to slow correction of sodium. Previous research has reported cases of extrapyramidal myelinolysis in malnourished and alcoholic patients who experienced neurological symptoms along with noninflammatory demyelination in the pons; the demyelination may even extend to extrapontine regions such as the basal ganglia, hippocampi, lateral geniculate bodies, cerebral white matter, and peripheral cortex [3]. EPM is seen in up to 10% of ODS cases [4].

EPM is characterized by tremor, ataxia, and other movement disorders including mutism, Parkinsonism, dystonia, and catatonia [5]. It is frequently linked to the fast correction of hyponatremia (sodium level rise of more than 12 mmol/day). The exact causes of ODS are unclear, although depletion of the adaptive process to prevent brain swelling leads to ODS. Brain shrinkage from redistribution of solutes following correction of hyponatremia causes tight junction disruption and blood-brain barrier disruption, which, in turn, causes oligodendrocyte damage and demyelination of neurons. Additionally, it has been demonstrated that the correction of hyponatremia results in the downregulation of a neutral amino acid transporter, which impairs cellular absorption of amino acids and increases cellular susceptibility to damage. An akinetic rigid state and postural dysfunctions are common manifestations in EPM, but secondary parkinsonism is rare [6].

The diagnosis of EPM is based primarily on clinical course, but demyelinating sites on MRI such as the cerebellum, thalamus, lateral geniculate body, and caudate nucleus confirm the diagnosis [7]. In our case, there were subtle hyperintensities in the caudate lobe.

Increasing plasma Na+ in asymptomatic patients should be done very gradually (0.5-1.0 mmol/h and up to 10-12 mmol/L over the first 24 hours). A relatively quick correction (1-2 mmol/L each hour for the first two to four hours or until seizures stop and up to 10-12 mmol/L throughout the first 24 hours) is advised for individuals with altered mental status and/or seizures [8].

A similar case to ours showed that rapid correction of sodium levels leads to EPM with parkinsonism features and was completely resolved with dopamine supplements [9]. Another case report, from Nepal, showed neuropsychiatric manifestation in a rapidly corrected hyponatremia. Following rapid correction, the patient developed features of parkinsonism such as tremors, mutism, whole-body rigidity, and sudden outbursts of laughter. She was treated with levodopa and carbidopa with anti-psychotic quetiapine, which showed improvement [6].

Our patient, who showed features of parkinsonism post-hyponatremia correction with subtle hyperintensities in the caudate lobe, thus confirming the diagnosis of EPM, showed improvement after starting the levodopa-carbidopa combination. At one-month follow-up, the patient showed great improvement, as she was able to walk, talk, and continue her daily activities.

## Conclusions

This is a case of extrapontine myelinolysis and rapid correction of hyponatremia. ODS can be treated with dopamine analogs like levodopa, and this case shows that the patient fully recovered after levodopa therapy. The guidelines for the treatment of severe hyponatremia suggest that we should administer 100 mL of 3% hypertonic saline up to three boluses with each infused over 10 minutes. These guidelines should be considered and reviewed, as bolus administration of hypertonic saline might cause ODS, which might be life-threatening. Physicians should be watchful for the rapid correction of hyponatremia. This case shows subtle hyperintensities in the caudate lobe of the brain, which are not very classic for extrapontine myelinolysis, but they can be diagnosed and treated on clinical signs and symptoms. Early and apt identification of extrapontine myelinolysis plays a pivotal role in managing this rare complication.
